# Secular polar motion observed by GRACE

**DOI:** 10.1007/s00190-021-01476-x

**Published:** 2021-03-22

**Authors:** Ki-Weon Seo, Jae-Seung Kim, Kookhyoun Youm, Jianli Chen, Clark R. Wilson

**Affiliations:** 1 Department of Earth Science Education, Seoul National University, Seoul, 08826 Republic of Korea; 2 Center for Space Research, University of Texas at Austin, Austin, TX 78759 USA; 3 Department of Geological Sciences, Jackson School of Geosciences, University of Texas at Austin, Austin, TX 78712 USA

**Keywords:** True polar wander, GIA, GRACE

## Abstract

A long-term drift in polar motion (PM) has been observed for more than a century, and Glacial Isostatic Adjustment (GIA) has been understood as an important cause. However, observed PM includes contributions from other sources, including contemporary climate change and perhaps others associated with Earth’s interior dynamics. It has been difficult to separate these effects, because there is considerable scatter among GIA models concerning predicted PM rates. Here we develop a new method to estimate GIA PM using data from the GRACE mission. Changes in GRACE degree 2, order 1 spherical harmonic coefficients are due both to GIA and contemporary surface mass load changes. We estimate the surface mass load contribution to degree 2, order 1 coefficients using GRACE data, relying on higher-degree GRACE coefficients that are dominantly affected by surface loads. Then the GIA PM trend is obtained from the difference between observed PM trend (which includes effects from GIA and surface mass loads) and the estimated PM trend mostly associated with surface mass loads. A previous estimate of the GIA PM trend from PM observations for the period 1900–1978 is toward 79.90° W at a speed of 3.53 mas/year (10.91 cm/year). Our new estimate for the GIA trend is in a direction of 61.77° W at a speed of 2.18 mas/year (6.74 cm/year), similar to the observed PM trend during the early twentieth century. This is consistent with the view that the early twentieth-century trend was dominated by GIA and that more recently there is an increasing contribution from contemporary surface mass load redistribution associated with climate change. Our GIA PM also agrees with the linear mean pole during 1900–2017. Contributions from other solid Earth process such as mantle convection would also produce a linear trend in PM and could be included in our GIA estimate.

## Introduction

Polar motion (PM), movement of Earth’s rotational axis relative to the crust, is a geophysical phenomenon excited by relative motion and mass redistribution within the Earth system. Sources include the atmosphere, hydrosphere, cryosphere and solid Earth (Gross 2007). PM observations and prediction (from geophysical data and models) provide a unique integrated view of Earth system changes. PM observations show an evident Chandler wobble, the Eulerian free wobble with about 14-month period. Annual and interannual PM variations are forced by relative motion of winds and ocean currents and mass redistribution of air and water (Gross 2007). Markowitz (1961) found quasi-periodic multi-decadal oscillations (Markowitz wobble) in PM, but their origin was unknown.

Recent studies have examined PM as a measure of contemporary climate change and found causes of interannual and longer period PM variations. For example, Youm et al. (2017) showed that interannual PM is driven by changes in terrestrial water storage, barometric pressure and ocean bottom pressure. At timescales of a decade and longer, PM reflects ice mass changes in polar ice sheets and mountain glaciers. The direction of PM drift changed around 2005, largely due to accelerated ice melting in Greenland (Chen et al. 2013), and additional changes in direction around 2011 were caused by terrestrial water storage sources (Adhikari and Ivins 2016). Identification of these contemporary sources has been enabled by entirely new capabilities to measure changes in Earth’s gravity field provided by the GRACE mission (Tapley et al. 2019).

A dominant contributor to a linear PM drift is recognized to be Glacial Isostatic Adjustment (GIA). GIA reflects redistribution of mass in the mantle, as a viscoelastic response to the last glacial maximum (e.g., Sabadini and Peltier 1981). Due to the long relaxation time of the mantle compared to the length of PM observations (120 years), the GIA effect will appear linear in time, with a constant speed and direction of average pole position during the last century. However, surface mass load changes associated with contemporary climate, including ice sheet melting, terrestrial water storage changes, and associated sea-level variation, will also contribute to the observed linear PM trend (Adhikari et al. 2018).

Recently, GRACE data were used to estimate the contemporary surface mass load contribution, and compared with PM observations, which involved adoption of a GIA model to remove this contribution and to correct GRACE data (Adhikari and Ivins 2016). Underlying this are assumptions about the accuracy of GIA models and dominance of GIA in the twentieth-century PM trend. However, as we will show later, there is large variability among GIA models, especially in predicted rates of Spherical Harmonic (SH) degree 2, order 1 coefficients (C_21_ and S_21_).

Therefore, accurate separation of the two sources (GIA and contemporary surface mass loads) in the PM trend (secular PM) has been difficult although it is critical to understanding the viscoelastic response of the mantle and rotational theories, as well as contemporary global change driven by a warming climate. In this study, we estimate separate contributions to secular PM associated with GIA and contemporary mass redistribution using GRACE (CSR RL06) degree 2, order 1 SH coefficients. We refer to observed coefficients as ($C_{21}^{{{\text{CSR}}}}$, $S_{21}^{{{\text{CSR}}}}$), which are the sum of GIA, ($C_{21}^{{{\text{GIA}}}}$, $S_{21}^{{{\text{GIA}}}}$), and contemporary surface mass load, ($C_{21}^{{{\text{LOAD}}}}$, $S_{21}^{{{\text{LOAD}}}}$), sources. The essential idea is that ($C_{21}^{{{\text{LOAD}}}}$, $S_{21}^{{{\text{LOAD}}}}$) can be estimated using higher-degree GRACE SH coefficients (Sun et al. 2016) in which surface mass load effects are dominant. In this way the PM trend associated with contemporary surface mass loads is obtained, and the GIA PM can be estimated using the difference between the observed PM trend (the sum of contemporary surface mass load and GIA) and the PM trend associated with contemporary surface mass loads alone. We find that GIA PM estimated here is different from current model predictions, which may indicate limited understanding of GIA and/or contributions of other solid Earth process such as mantle convection.

## Separating $(C_{21}^{{{\text{LOAD}}}}$, $S_{21}^{{{\text{LOAD}}}} )$ from $(C_{21}^{{{\text{CSR}}}}$, $S_{21}^{{{\text{CSR}}}} )$

**Fig. 1:**
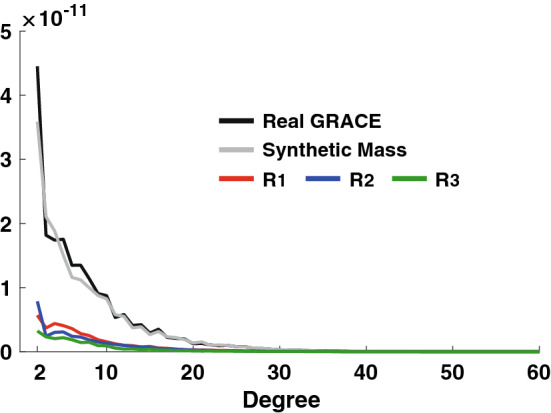
Degree amplitudes of real (black) and synthetic (gray) GRACE data. Only the linear trend in each SH coefficient is considered. R_1_, R_2_ and R_3_ are degree amplitudes of GIA model differences

It has been a common practice to correct GRACE SH coefficients using GIA models. These corrected SH coefficients are presumed to be due to contemporary surface mass loads, but may also include residual GIA effects due to imperfect GIA models. An estimate of the size of these residuals can be understood by examining variations among GIA models, which provide predictions of SH coefficient time rates of change. We first consider three models: A13 (A et al. 2013), Purcell16 (Paulson et al. 2007) and Peltier18 (Peltier et al. 2018). Differences between all pairs, A13–Purcell16 (R_1_), A13–Peltier18 (R_2_) and Peltier18–Purcell16 (R_3_), are the red, blue and green lines in Fig. 1, showing degree amplitudes of R_1_, R_2_ and R_3_, respectively. These GIA model differences can be taken as measures of model error, including different realizations of rotational theories. We compare these GIA model errors with surface mass load signals. Figure 1 shows CSR RL06 RMS linear trend amplitudes in terms of degree variances from January 2003 to December 2015. RL06 GRACE data were processed by applying a decorrelation filter (Swenson and Wahr 2006), 500 km Gaussian smoothing, and then removing GIA effects using the A13 model. The GRACE surface mass load signal (black line) is much larger than the apparent GIA model error. However, the largest GIA model error in degree 2 is associated with degree 2, order 1, and thus $(C_{21}^{{{\text{LOAD}}}}$, $S_{21}^{{{\text{LOAD}}}} )$ would suffer from relatively large uncertainties if estimated from the difference between $(C_{21}^{{{\text{CSR}}}}$, $S_{21}^{{{\text{CSR}}}} )$ and GIA models with erroneous degree 2, order 1 coefficients.

Synthetic data can be useful to understand uncertainty in $(C_{21}^{{{\text{LOAD}}}}$, $S_{21}^{{{\text{LOAD}}}} )$ associated with unmodeled GIA effects. A synthetic GRACE data set is constructed using various numerical climate models and observations of surface mass load changes. For ice mass changes in Greenland and Antarctica, we use GRACE estimates after correcting spatial leakage effects (Kim et al. 2019). Ice mass loss over mountain glaciers is included by adding linear trends over glaciated regions from a glacier mass balance model (Zemp et al. 2019). Terrestrial water storage (TWS) change is taken from predictions of the global land data assimilation system (GLDAS) (Rodell et al. 2004). Total ocean mass change is the negative of all changes over land, and its distribution over the oceans is calculated by imposing Self Attraction and Loading (SAL) (Farrell and Clark 1976). We add an estimate of GRACE error to the synthetic data, equal to the difference between observed and smoothed SH coefficients, with a correction for residual signals using empirical orthogonal function analysis (Eom et al. 2017). The synthetic data are then subjected to decorrelation and 500 km Gaussian smoothing filters. The gray line in Fig. 1 shows linear trend amplitudes for the synthetic data as a function of SH degree, confirming similarity with GRACE CSR RL06.

**Fig. 2:**
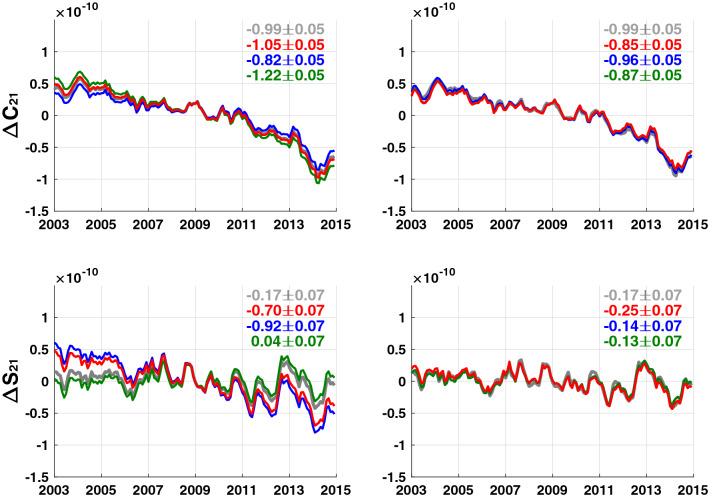
Left panels: time series of C_21_ and S_21_ in synthetic GRACE data. Gray lines are ‘true’ coefficients, and red, blue and green lines are coefficients contaminated by GIA error of R_1_, R_2_ and R_3_, respectively. Right panels: similar time series of the left panels. C_21_ and S_21_ are estimated coefficients by suppressing residual GIA error

We add GIA model differences (R_1_, R_2_ and R_3_) to the synthetic data to simulate GIA model error. Left panels of Fig. 2 show GIA errors in the synthetic C_21_ and S_21_ SH coefficients. Gray lines in the left panels represent ‘true’ $(C_{21}^{{{\text{LOAD}}}}$, $S_{21}^{{{\text{LOAD}}}} )$ coefficients from the synthetic data, and red, blue and green lines are ‘observed’ $(C_{21}^{{{\text{LOAD}}}}$, $S_{21}^{{{\text{LOAD}}}} )$ contaminated by R_1_, R_2_ and R_3_ GIA model errors. It is clear that GIA model errors dominate trends in S_21_ SH coefficients, indicating large error in GIA model and resulting uncertainty in the estimation of $S_{21}^{{{\text{LOAD}}}}$.

Here we develop a new method to estimate $(C_{21}^{{{\text{LOAD}}}}$, $S_{21}^{{{\text{LOAD}}}} )$ by reducing GIA residuals as shown in the left panels of Fig. 2. We estimate $(C_{21}^{{{\text{LOAD}}}}$, $S_{21}^{{{\text{LOAD}}}} )$ from synthetic GRACE data using an idea developed by Sun et al. (2016). They estimated degree-1 and degree-2, order-0 SH coefficients simultaneously by modifying an earlier approach to find degree-1 SH coefficients (Swenson et al. 2008). We extend the method to estimate degree-2, order-1 coefficients, which depend on higher degrees and orders, so should be relatively uncontaminated by GIA model errors, as evident in Fig. 1.

Following Sun et al. (2016), the six coefficients ($C_{10}^{{{\text{LOAD}}}}$, $C_{11}^{{{\text{LOAD}}}}$, $S_{11}^{{{\text{LOAD}}}}$) and ($C_{20}^{{{\text{LOAD}}}}$, $C_{21}^{{{\text{LOAD}}}}$, $S_{21}^{{{\text{LOAD}}}}$) satisfy the following simultaneous linear equations,

1 $$ \begin{gathered} \left[ {\begin{array}{*{20}c} {C_{10}^{{{\text{ocean}}}} } \\ {C_{11}^{{{\text{ocean}}}} } \\ {S_{11}^{{{\text{ocean}}}} } \\ {C_{20}^{{{\text{ocean}}}} } \\ {C_{21}^{{{\text{ocean}}}} } \\ {S_{21}^{{{\text{ocean}}}} } \\ \end{array} } \right] = \left[ {\begin{array}{*{20}l} {I_{10C}^{10C} } \hfill & {I_{11C}^{10C} } \hfill & {I_{11S}^{10C} } \hfill & {I_{20C}^{10C} } \hfill & {I_{21C}^{10C} } \hfill & {I_{21S}^{10C} } \hfill \\ {I_{10C}^{11C} } \hfill & {I_{11C}^{11C} } \hfill & {I_{11S}^{11C} } \hfill & {I_{20C}^{11C} } \hfill & {I_{21C}^{11C} } \hfill & {I_{21S}^{11C} } \hfill \\ {I_{10C}^{11S} } \hfill & {I_{11C}^{11S} } \hfill & {I_{11S}^{11S} } \hfill & {I_{20C}^{11S} } \hfill & {I_{21C}^{11S} } \hfill & {I_{21S}^{11S} } \hfill \\ {I_{10C}^{20C} } \hfill & {I_{11C}^{20C} } \hfill & {I_{11S}^{20C} } \hfill & {I_{20C}^{20C} } \hfill & {I_{21C}^{20C} } \hfill & {I_{21S}^{20C} } \hfill \\ {I_{10C}^{21C} } \hfill & {I_{11C}^{21C} } \hfill & {I_{11S}^{21C} } \hfill & {I_{20C}^{21C} } \hfill & {I_{21C}^{21C} } \hfill & {I_{21S}^{21C} } \hfill \\ {I_{10C}^{21S} } \hfill & {I_{11C}^{21S} } \hfill & {I_{11S}^{21S} } \hfill & {I_{20C}^{21S} } \hfill & {I_{21C}^{21S} } \hfill & {I_{21S}^{21S} } \hfill \\ \end{array} } \right]\left[ {\begin{array}{*{20}c} {C_{10}^{{{\text{LOAD}}}} } \\ {C_{11}^{{{\text{LOAD}}}} } \\ {S_{11}^{{{\text{LOAD}}}} } \\ {C_{20}^{{{\text{LOAD}}}} } \\ {C_{21}^{{{\text{LOAD}}}} } \\ {S_{21}^{{{\text{LOAD}}}} } \\ \end{array} } \right] + \left[ {\begin{array}{*{20}c} {G_{10C}^{{}} } \\ {G_{11C}^{{}} } \\ {G_{11S}^{{}} } \\ {G_{20C}^{{}} } \\ {G_{21C}^{{}} } \\ {G_{21S}^{{}} } \\ \end{array} } \right] \hfill \\ \hfill \\ \end{gathered} $$

Here values in the column vector $$\left[ {\begin{array}{*{20}c} {C_{10}^{{{\text{ocean}}}} } & {C_{11}^{{{\text{ocean}}}} } & {\begin{array}{*{20}c} {S_{11}^{{{\text{ocean}}}} C_{20}^{{{\text{ocean}}}} } & {C_{21}^{{{\text{ocean}}}} } & {S_{21}^{{{\text{ocean}}}} } \\ \end{array} } \\ \end{array} } \right]^{{\text{T}}}$$ represent water mass redistribution over the oceans. This method is valid because ocean mass load is determined by terrestrial mass load via water mass exchange between land and oceans. Other effects such as ocean dynamics and barometric pressure cannot be included in this equation but can possibly be considered later for the full SH coefficients including all effects (Swenson et al. 2008). Previously, the method was developed to estimate degree-1 SH coefficients assuming that ocean mass load was the uniformly distributed negative of total terrestrial water mass change (Swenson et al. 2008). Because over the oceans the load adjusts to conform to a changed geoid, it was refined later to include the SAL effect (Sun et al. 2016). However, a limitation of their refinement was to correct spatial leakage effects empirically. The correction of leakage effects is important in Eq. (1) because it separates land and ocean mass load changes. In this study $$\left[ {\begin{array}{*{20}c} {C_{10}^{{{\text{ocean}}}} } & {C_{11}^{{{\text{ocean}}}} } & {\begin{array}{*{20}c} {S_{11}^{{{\text{ocean}}}} C_{20}^{{{\text{ocean}}}} } & {C_{21}^{{{\text{ocean}}}} } & {S_{21}^{{{\text{ocean}}}} } \\ \end{array} } \\ \end{array} } \right]^{{\text{T}}}$$ were predicted by SAL after correcting GRACE data for spatial leakage into the oceans using forward modeling (FM) (Jeon et al. 2018).

The $\overline{I}$ matrix is determined from the ocean function, $\vartheta \left( {\theta ,\phi } \right)$, equal to zeros on land and ones over the oceans

2 $$ \overline{I} = \frac{1}{4\pi } \smallint \overline{U}\overline{U}^{{\text{T}}} \vartheta \left( {\theta , \phi } \right) {\text{d}}\Omega $$

where $\overline{U}$ is given by

3 $$ \overline{U} = \left[ {\begin{array}{*{20}c} {U_{10C}^{{}} } & {U_{11C}^{{}} } & {\begin{array}{*{20}c} {U_{11S}^{{}} } & {U_{20C}^{{}} U_{21C}^{{}} } & {U_{21S}^{{}} } \\ \end{array} } \\ \end{array} } \right]^{{\text{T}}} $$

whose components are

4 $$ U_{lm\psi } = \tilde{P}_{lm} \left( {\cos \theta } \right)\left\{ {\begin{array}{*{20}c} {\cos \left( {m\phi } \right) \left( { \psi = C} \right)} \\ {\sin \left( {m\phi } \right) \left( { \psi = S} \right)} \\ \end{array} } \right. $$

Here, $\theta$ and $\phi$ are colatitude and longitude, and $\tilde{P}_{lm}$ are normalized associated Legendre functions. The $\overline{G}$ vector consists of $\overline{U}$ and global SH coefficients estimated after FM:

5 $$ G_{lm\psi } = \frac{1}{4\pi }\int {U_{lm\psi } } \vartheta \left( {\theta , \phi } \right)\sum\limits_{{l^{\prime } = 2}}^{\infty } {\sum\limits_{{m^{\prime } = 0}}^{{l^{\prime } }} {\tilde{P}_{{l^{\prime } m^{\prime } }} } } \left( {\cos \theta } \right)\left\{ {C_{{l^{\prime } m^{\prime } }}^{{{\text{LOAD}}}} \cos \left( {m^{\prime } \phi } \right) + S_{{l^{\prime } m^{\prime } }}^{{{\text{LOAD}}}} \sin \left( {m^{\prime } \phi } \right)} \right\} {\text{d}}\Omega $$

In Eq. (5), $(C_{20}^{{{\text{LOAD}}}} ,C_{21}^{{{\text{LOAD}}}}$, $S_{21}^{{{\text{LOAD}}}}$) are initially zero. We iteratively solve Eq. (1), updating $$\left[ {\begin{array}{*{20}c} {C_{10}^{{{\text{ocean}}}} } & {C_{11}^{{{\text{ocean}}}} } & {\begin{array}{*{20}c} {S_{11}^{{{\text{ocean}}}} C_{20}^{{{\text{ocean}}}} } & {C_{21}^{{{\text{ocean}}}} } & {S_{21}^{{{\text{ocean}}}} } \\ \end{array} } \\ \end{array} } \right]^{{\text{T}}}$$ until $${ }\left[ {\begin{array}{*{20}c} {C_{10}^{{{\text{LOAD}}}} } & {C_{11}^{{{\text{LOAD}}}} } & {\begin{array}{*{20}c} {S_{11}^{{{\text{LOAD}}}} C_{20}^{{{\text{LOAD}}}} } & {C_{21}^{{{\text{LOAD}}}} } & {S_{21}^{{{\text{LOAD}}}} } \\ \end{array} } \\ \end{array} } \right]^{{\text{T}}}$$ converge. The resulting degree-1 and degree-2 coefficients $( C_{20}^{{{\text{LOAD}}}} ,C_{21}^{{{\text{LOAD}}}}$, $S_{21}^{{{\text{LOAD}}}}$) should reflect mainly contemporary surface water and ice mass changes including minor GIA model errors because they are determined from higher-degree coefficients, as shown in Fig. 1, in which contemporary mass change effects are much larger than likely GIA model uncertainty. After iterative solutions to Eq. (1), resulting coefficient time series are shown in the right panels of Fig. 2. The three different estimates agree very well with one another and with the ‘true’ time series. This verifies the effectiveness of this approach to correcting GIA model error in estimated $(C_{21}^{{{\text{LOAD}}}}$, $S_{21}^{{{\text{LOAD}}}}$).

**Fig. 3:**
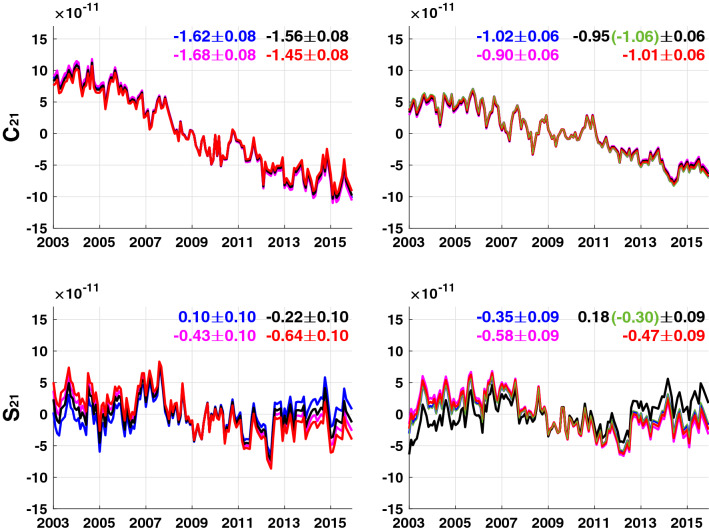
Left panels: time series of C_21_ and S_21_ in GRACE data after GIA correction using four GIA models. Blue, magenta, black and red lines correspond to GIA models A13, Purcell16, Caron18 and Peltier18, respectively. Right panels: similar to the left panels using C_21_ and S_21_ coefficients with suppressed GIA errors. Green lines replace S_31_ in Caron18 with that from Peltier18

We apply the same method to real GRACE data. Figure 3 shows time series of conventional GRACE $(C_{21}^{{{\text{LOAD}}}}$, $S_{21}^{{{\text{LOAD}}}} )$ in the left panels, using a total of four different GIA models, including the previous three, plus Caron18 (Caron et al. 2018). Blue, magenta, black and red lines show conventional GRACE $(C_{21}^{{{\text{LOAD}}}}$, $S_{21}^{{{\text{LOAD}}}} )$ using models of A13, Purcell16, Caron18 and Peltier18, respectively. As in the synthetic experiment in Fig. 2, trend differences are evident in $S_{21}^{{{\text{LOAD}}}}$. Figures 2 and 3 together show the limitations of GIA models in separating $(C_{21}^{{{\text{LOAD}}}}$,$S_{21}^{{{\text{LOAD}}}} )$ from ($C_{21}^{{{\text{CSR}}}}$, $S_{21}^{{{\text{CSR}}}}$).

Similar results are obtained using satellite laser ranging (SLR) SH coefficients ($C_{21}^{{{\text{SLR}}}}$, $S_{21}^{{{\text{SLR}}}}$) (Cheng et al. 2013) in place of GRACE $(C_{21}^{{{\text{LOAD}}}}$, $S_{21}^{{{\text{LOAD}}}} )$. Both GRACE and SLR sense GIA and surface mass loads, so estimated $(C_{21}^{{{\text{LOAD}}}}$, $S_{21}^{{{\text{LOAD}}}} )$ from either SLR or GRACE share common GIA model uncertainty. For example, trends in $(C_{21}^{{{\text{LOAD}}}}$, $S_{21}^{{{\text{LOAD}}}} )$ estimated from ($C_{21}^{{{\text{SLR}}}}$, $S_{21}^{{{\text{SLR}}}}$) (using Peltier18) are (− 1.41 × 10^–11^, − 0.71 × 10^–11^), very close to GRACE values in Fig. 3 (− 1.45 × 10^–11^, − 0.64 × 10^–11^, red lines in left panels).

The four $C_{21}^{{{\text{LOAD}}}}$ time series (Fig. 3 left panel) have similar trends near $- 1.5 \times 10^{ - 11}$ year^−1^, so differences among GIA model predictions for this coefficient are small. Trends in the four $C_{21}^{{{\text{LOAD}}}}$ estimated here (right panel) are similar, but smaller, about $- 1.0 \times 10^{ - 11}$ year^−1^. Uncertainties in GIA models at higher SH degrees will affect C_21_ estimation. However, as shown in Fig. 1, surface mass loads tend to be much larger than differences among GIA models, and thus the higher SH degree uncertainties in GIA models would not be a cause of the trend change. Instead we suspect the change may be due to a common C_21_ bias in GIA models.

**Fig. 4:**
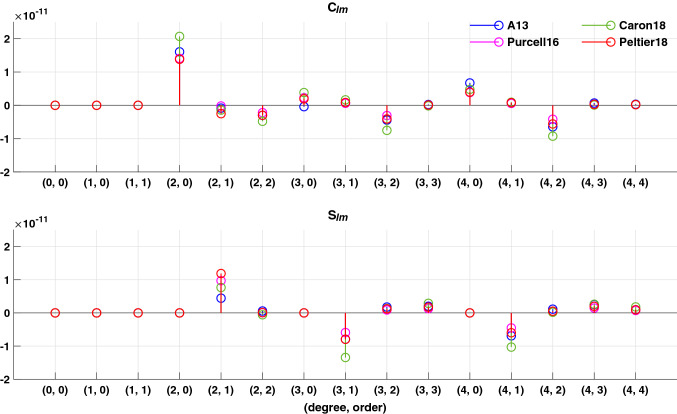
GIA predictions up to degree and order 4 from four GIA models

Estimated $S_{21}^{{{\text{LOAD}}}}$ (right panel) trends are similar for most GIA models, with the exception of Caron18 (black). Caron18 values for $S_{31}$ rates are large relative to the other models (Fig. 4). This will affect the $(C_{21}^{{{\text{LOAD}}}}$, $S_{21}^{{{\text{LOAD}}}}$) estimate. If we replace $S_{31}$ in Caron18 with S_31_ from Peltier18, and then estimate $(C_{21}^{{{\text{LOAD}}}}$, $S_{21}^{{{\text{LOAD}}}} )$ again, the results (green lines and numbers in Fig. 3 right panel) are similar to those of the other models.

We have estimated SH degree 2, order 1 coefficients associated with contemporary surface mass load, $(C_{21}^{{{\text{LOAD}}}}$, $S_{21}^{{{\text{LOAD}}}} )$, by reducing GIA model errors. There were large trend differences in $(C_{21}^{{{\text{LOAD}}}}$, $S_{21}^{{{\text{LOAD}}}} )$ when they were obtained (in the conventional way) using the difference between observed degree 2, order 1 SH coefficients (from GRACE or SLR) and a GIA model prediction. Now trends in estimated $(C_{21}^{{{\text{LOAD}}}}$, $S_{21}^{{{\text{LOAD}}}} )$ are similar regardless of GIA model choice. The experiment with synthetic data confirmed the validity of this approach to $(C_{21}^{{{\text{LOAD}}}}$, $S_{21}^{{{\text{LOAD}}}} )$ estimation.

## Climate-driven PM trend

Climate-driven PM trends, largely caused by melting ice in polar ice sheets and mountain glaciers, terrestrial water storage change and sea level rise, can be estimated using least square linear fits to coefficient time series shown in the right panel of Fig. 3. SH coefficient rates $(\dot{C}_{21}^{{{\text{LOAD}}}} ,\dot{S}_{21}^{{{\text{LOAD}}}} )$ are proportional to PM rates via the relationship (e.g., Lambeck 1980):

6 $$ \left( {\begin{array}{*{20}c} {\dot{m}_{1}^{{{\text{LOAD}}}} } \\ {\dot{m}_{2}^{{{\text{LOAD}}}} } \\ \end{array} } \right) \approx R\frac{{ - Ma^{2} \sqrt {5/3} }}{{\left( {C - A} \right)}}\left( {\begin{array}{*{20}c} {\dot{C}_{21}^{{{\text{LOAD}}}} } \\ {\dot{S}_{21}^{{{\text{LOAD}}}} } \\ \end{array} } \right) $$

$(\dot{m}_{1}^{{{\text{LOAD}}}}$, $\dot{m}_{2}^{{{\text{LOAD}}}} )$ are PM components (in arcsec) along the Greenwich meridian and $90^{^\circ }$ east of Greenwich, respectively, and *R* is a conversion factor from radians to arcsec. PM due to surface mass loads deforms the solid Earth and oceans via the pole tide, requiring adjustment to degree 2, order 1 SH coefficients (Wahr et al. 2015):

7 $$ \left( {\begin{array}{*{20}c} {\dot{C}_{21}^{{{\text{PT}}}} } \\ {\dot{S}_{21}^{{{\text{PT}}}} } \\ \end{array} } \right) = \left( {\begin{array}{*{20}c} { - 1.551 \times 10^{ - 9} } \\ {0.021 \times 10^{ - 9} } \\ \end{array} } \right)\dot{m}_{1}^{{{\text{LOAD}}}} + \left( {\begin{array}{*{20}c} { - 0.012 \times 10^{ - 9} } \\ { - 1.505 \times 10^{ - 9} } \\ \end{array} } \right)\dot{m}_{2}^{{{\text{LOAD}}}} $$

The deformation associated with the pole tide is equivalent to an apparent surface mass load change, which amplifies PM and, in turn, deforms the Earth. This feedback between pole tide and PM has a net effect $\left( {\dot{C}_{21}^{{{\text{PT}}}} ,\dot{S}_{21}^{{{\text{PT}}}} } \right)$ that can be estimated iteratively using Eqs. (6) and (7). An iterative solution converged after 5 iterations. The degree 2, order 1 SH coefficients including effects from both surface mass load and the resulting pole tide,$\left( {\dot{C}_{21}^{{{\text{LOAD}} + {\text{PT}}}} ,\dot{S}_{21}^{{{\text{LOAD}} + {\text{PT}}}} } \right)$, are simply the sum of $(\dot{C}_{21}^{{{\text{LOAD}}}} ,\dot{S}_{21}^{{{\text{LOAD}}}} )$ and $\left( {\dot{C}_{21}^{{{\text{PT}}}} ,\dot{S}_{21}^{{{\text{PT}}}} } \right)$. Resulting polar motion components $(\dot{m}_{1}^{{{\text{LOAD}} + {\text{PT}}}}$, $\dot{m}_{2}^{{{\text{LOAD}} + {\text{PT}}}} )$ are proportional to these.

Alternatively, we can estimate $(\dot{m}_{1}^{{{\text{LOAD}} + {\text{PT}}}}$, $\dot{m}_{2}^{{{\text{LOAD}} + {\text{PT}}}} )$ by modifying Eq. (6) to include the pole tide effect:

8 $$ \left( {\begin{array}{*{20}c} {\dot{m}_{1}^{{{\text{LOAD}} + {\text{PT}}}} } \\ {\dot{m}_{2}^{{{\text{LOAD}} + {\text{PT}}}} } \\ \end{array} } \right) \approx R\frac{{ - Ma^{2} \sqrt {5/3} }}{{\left( {C - A} \right)}}\left( {\begin{array}{*{20}c} {\dot{C}_{21}^{{{\text{LOAD}}}} } \\ {\dot{S}_{21}^{{{\text{LOAD}}}} } \\ \end{array} } \right) + R\frac{{ - Ma^{2} \sqrt {5/3} }}{{\left( {C - A} \right)}}\left( {\begin{array}{*{20}c} {\dot{C}_{21}^{{{\text{PT}}}} } \\ {\dot{S}_{21}^{{{\text{PT}}}} } \\ \end{array} } \right) $$

Combining Eqs. (7) and (8) and ignoring minor effects of an elasticity in Eq. (7), estimated PM due to surface mass loads and accompanying pole tide can be directly obtained by

9 $$ \left( {\begin{array}{*{20}c} {\dot{m}_{1}^{{{\text{LOAD}} + {\text{PT}}}} } \\ {\dot{m}_{2}^{{{\text{LOAD}} + {\text{PT}}}} } \\ \end{array} } \right) \approx R\frac{{ - Ma^{2} \sqrt {5/3} }}{{\left( {C - A} \right)}}\left( {\begin{array}{*{20}c} {\dot{C}_{21}^{{{\text{LOAD}}}} } \\ {\dot{S}_{21}^{{{\text{LOAD}}}} } \\ \end{array} } \right) + R\frac{{ - Ma^{2} \sqrt {5/3} }}{{\left( {C - A} \right)}}\left( {\begin{array}{*{20}c} { - 1.551 \times 10^{ - 9} } \\ { - 1.505 \times 10^{ - 9} } \\ \end{array} } \right)\left( {\begin{array}{*{20}c} {\dot{m}_{1}^{{{\text{LOAD}} + {\text{PT}}}} } \\ {\dot{m}_{2}^{{{\text{LOAD}} + {\text{PT}}}} } \\ \end{array} } \right) $$

The iterative and direct methods provide almost identical estimates of $(\dot{m}_{1}^{{{\text{LOAD}} + {\text{PT}}}}$, $\dot{m}_{2}^{{{\text{LOAD}} + {\text{PT}}}} )$.

**Fig. 5:**
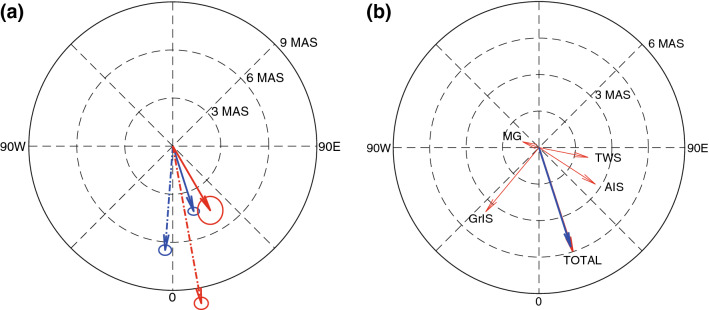
**a** Blue arrow shows $\left( {\dot{m}_{1}^{{{\text{LOAD}} + {\text{PT}}}} ,\dot{m}_{2}^{{{\text{LOAD}} + {\text{PT}}}} } \right)$ from $\left( {\dot{C}_{21}^{{{\text{LOAD}}}} ,\dot{S}_{21}^{{{\text{LOAD}}}} } \right)$ estimated by suppressing A13 error. Dashed-blue arrow is similar to the blue arrow except CSR RL06 is corrected with A13 (without error suppression). Red arrow shows $\left( {\dot{m}_{1}^{{{\text{LOAD}} + {\text{PT}}}} ,\dot{m}_{2}^{{{\text{LOAD}} + {\text{PT}}}} } \right)$ from a previous study (Adhikari and Ivins 2016). Dashed-red arrow is similar to the dashed-blue arrow except using CSR RL05. **b** Thin red arrows show each contributor to $\left( {\dot{m}_{1}^{{{\text{LOAD}} + {\text{PT}}}} ,\dot{m}_{2}^{{{\text{LOAD}} + {\text{PT}}}} } \right)$. Blue arrow is the same as in **a**. The sum of each contribution (red arrow) is almost identical to $\left( {\dot{m}_{1}^{{{\text{LOAD}} + {\text{PT}}}} ,\dot{m}_{2}^{{{\text{LOAD}} + {\text{PT}}}} } \right)$ (blue arrow) estimated from $\left( {\dot{C}_{21}^{{{\text{LOAD}}}} ,\dot{S}_{21}^{{{\text{LOAD}}}} } \right)$

Using the four GIA error corrected time series as in the right panel of Fig. 3, we obtain 4 values for $(\dot{C}_{21}^{{{\text{LOAD}}}}$, $\dot{S}_{21}^{{{\text{LOAD}}}} )$ to obtain four PM trends, $(\dot{m}_{1}^{{{\text{LOAD}} + {\text{PT}}}}$, $\dot{m}_{2}^{{{\text{LOAD}} + {\text{PT}}}} )$. Because effects from atmospheric pressure and ocean bottom pressure have been removed using numerical models in GRACE data processing (Bettadpur 2018), we restore those model values to obtain total $(\dot{m}_{1}^{{{\text{LOAD}} + {\text{PT}}}}$, $\dot{m}_{2}^{{{\text{LOAD}} + {\text{PT}}}} )$ vectors. Adding atmospheric and ocean bottom pressure has a small effect, because water is the dominant source of long-term mass redistribution. An example of one of the four PM trends is shown in Fig. 5a. The dashed-blue arrow in Fig. 5a is the apparent PM trend estimated by $(\dot{C}_{21}^{{{\text{CSR}}}}$, $\dot{S}_{21}^{{{\text{CSR}}}} )$ after the GIA effect is removed using A13. Therefore, the arrow indicates the PM trend from both contemporary mass loads and residual GIA effects not removed by A13. On the other hand, the blue arrow nominally represents PM trends, $\left( {\dot{m}_{1}^{{{\text{LOAD}} + {\text{PT}}}} ,\dot{m}_{2}^{{{\text{LOAD}} + {\text{PT}}}} } \right),$ due to the contemporary surface mass load realized by $(\dot{C}_{21}^{{{\text{LOAD}}}}$, $\dot{S}_{21}^{{{\text{LOAD}}}} )$ after suppressing A13 error. The two arrows are different due to residual GIA not corrected by A13. From Fig. 3, it is clear that trends using the other three models are similar, including that associated with modified Caron18. Ellipses represent 95% confidence intervals for PM trends, based upon nominal uncertainty in linear trend fits.

Our new estimate of PM trend due to surface mass loads and pole tide (blue arrow in Fig. 5a) can be compared with the estimate of Adhikari and Ivins (2016) (Fig. 5a, red arrow) based on CSR RL05 and A13 for the same period. Similar trend estimated from CSR RL05 C_21_ and S_21_ (with A13) is shown by the dashed-red arrow. The dashed-blue and dashed-red arrows include common effects of surface mass loads and A13 uncertainties, but differ due to different pole tide corrections in CSR RL06 and RL05. The dashed-red arrow (the difference between CSR RL05 C_21_ and S_21_ with A13) and the red arrow from Adhikari and Ivins (2016) should be identical, given that the same GRACE data and A13 model were used. Instead, there is a large difference. The Adhikari and Ivins (2016) red arrow is rather similar to our blue arrow, in which A13 error was suppressed. We suspect the Adhikari and Ivins (2016) estimate may have effectively suppressed A13 error by separating trend contributions from Antarctic and Greenland Ice Sheets (AIS, GrIS), Mountain Glaciers (MG) and Terrestrial Water Storage (TWS). Adhikari and Ivins (2016) used empirical scale factors (e.g., Landerer and Swenson 2012) to deal with spatial leakage instead of our approach using FM. The ocean mass contribution was estimated considering SAL, as in this study. For example, the Adhikari and Ivins (2016) estimate for the GrIS contribution used leakage corrected regional mass fields plus consequent ocean mass change. Because regional surface mass loads (individual AIS, GrIS, TWS and MG contributions) are dominated by higher-degree SH coefficients, separate treatment of these regional contributions would probably also suppress GIA error.

There is a large difference between the dashed-red line (CSR RL05 C_21_ and S_21_; with A13) and red arrows (sum of CSR RL05 AIS, GrIS, MG and TWS; correction by A13) from Adhikari and Ivins (2016). Our estimate is consistent with a sum of each PM contribution shown by thin red arrows and their sum in Fig. 5b. The TWS trend is taken to be the residual after removing GRACE (FM corrected) AIS and GrIS trends and an MG trend using the model of Zemp et al. (2019). Trends due to atmospheric and ocean bottom pressure are also included but are omitted from the figure because they are small. The thick red arrow is the sum of all components and is almost identical to the thick blue arrow (same as Fig. 5a, but note that scales are different).

## GIA PM trend

**Fig. 6:**
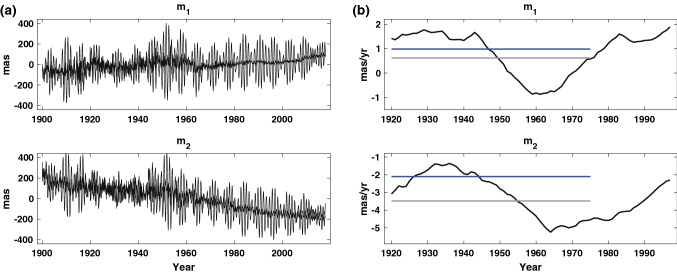
**a** PM observations (gray) from 1900 to 2017 and excitations (black) after Chandler wobble correction. **b** Variations of 40-year mean PM trends (black). Gray horizontal lines are observed PM trends during 1900–1978. Blue horizontal lines are estimated GIA PM trends in this study

It has been assumed that the long-term PM trend is dominantly affected by GIA (e.g., Wahr et al. 2015; Adhikari and Ivins 2016), but both GIA and climate-driven contemporary surface mass loads may contribute (Mitrovica et al. 2015; Munk 2002). Industrial-era warming might have commenced since the mid-nineteenth century (Abram et al. 2016), so global mass redistribution related to ice melting and sea-level rise may have become significant during the twentieth century. Mountain glacier retreat and subsequent sea-level rise have been observed since the early twentieth century (e.g., Parkes and Marzeion (2018)). One may expect that PM trends would be affected by these sources. Figure 6a shows PM observations from 1900 to 2017 (gray) and excitation (black) after removing the Chandler wobble resonance using the linear filter of Wilson (1985). The trends in ($m_{1}$, $m_{2}$) have long been recognized, as well as multi-decadal variability, which has in recent studies been associated with surface mass redistribution (Chen et al. 2013; Adhikari and Ivins 2016). Figure 6b shows changes in PM trend rates using a running 40-year window fit to the excitation series of Fig. 6a. For example, the rate for 1920 is determined from the polar motion time series from 1900 to 1940. Data prior to 1900 (when the International Latitude Service was founded) are not used due to poorer quality. There is considerable variability evident in PM trends. The $m_{1}$ trend changes little between 1920 and 1940, but after 1940 a negative trend continues until 1960 and then becomes positive. The $m_{2}$ trend between 1920 and 1960 varies from about − 1 to − 5 mas/year. If twentieth-century PM trends were dominated by GIA, they should be approximately constant, except for errors in the data. Separating the two effects, contemporary surface mass load and GIA, does not seem possible using only the historical PM data.

We can estimate PM associated with GIA $\left( {\dot{m}_{1}^{{{\text{GIA}}}} ,\dot{m}_{2}^{{{\text{GIA}}}} } \right)$ using the difference between $(\dot{m}_{1}^{{{\text{LOAD}} + {\text{PT}}}}$, $\dot{m}_{2}^{{{\text{LOAD}} + {\text{PT}}}} )$ and $(\dot{m}_{1}^{{{\text{OBS}}}} ,\dot{m}_{2}^{{{\text{OBS}}}} )$ = $\left( {5.04 {\text{mas}}/{\text{year}},\; - \,0.66 {\text{mas}}/{\text{year}}} \right)$, observed PM trends during the GRACE era (January 2003–December 2015). Because $(\dot{m}_{1}^{{{\text{OBS}}}} ,\dot{m}_{2}^{{{\text{OBS}}}} )$ includes effects of GIA, surface mass load and pole tide, the difference, $(\dot{m}_{1}^{{{\text{OBS}}}} ,\dot{m}_{2}^{{{\text{OBS}}}} )$ − $(\dot{m}_{1}^{{{\text{LOAD}} + {\text{PT}}}}$, $\dot{m}_{2}^{{{\text{LOAD}} + {\text{PT}}}} )$, is the GIA PM trend, $\left( {\dot{m}_{1}^{{{\text{GIA}}}} ,\dot{m}_{2}^{{{\text{GIA}}}} } \right)$.

**Fig. 7:**
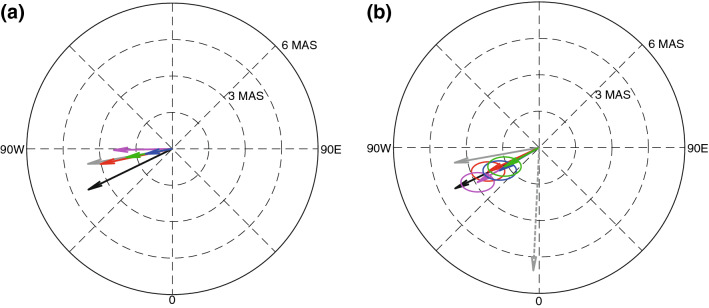
**a** Predictions of GIA PM from GIA models of A13 (blue), Purcell16 (magenta), Caron18 (green) and Peltier18 (red). **b** Estimated GIA PMs, $\left( {\dot{m}_{1}^{{{\text{GIA}}}} ,\dot{m}_{2}^{{{\text{GIA}}}} } \right)$, using GRACE data after the corrections of GIA model errors in A13 (blue), Purcell16 (magenta), Caron18 (green) and Peltier18 (red). Gray and black arrows in **a** and **b** are observed PM trend during 1900–1978 and the 1900–2017 (linear mean pole), respectively. The dashed-gray arrow in **b** is observed PM trend for January 2003 to December 2015

Figure 7a shows the great variability of GIA PM trends among the four models, without error correction. Trend directions from GIA models of A13 (blue), Caron18 (green) and Peltier18 (red) are similar to the 1900–1978 average (gray), but their speeds differ significantly. Wahr et al. (2015) assumed that the gray line is the GIA PM trend. The trend direction from Purcell16 (magenta) is rather different from others, while the trend from Peltier18 is very close to the 1900–1978 average. Furthermore, the four predictions and the 1900–1978 average are clearly different from the linear mean pole (black), the average PM trend for 1900–2017, which is the currently adopted GIA PM trend (Petit and Luzum 2010). These discrepancies among model predictions and long-term PM observations confirm larger uncertainties in GIA PM. Figure 7b shows four GIA PM trends estimated in this study by the difference between $\left( {\dot{m}_{1}^{{{\text{OBS}}}} ,\dot{m}_{2}^{{{\text{OBS}}}} } \right)$ (dashed-gray) and $\left( {\dot{m}_{1}^{{{\text{LOAD}} + {\text{PT}}}} ,\dot{m}_{2}^{{{\text{LOAD}} + {\text{PT}}}} } \right)$. Blue, magenta, green and red arrows show values using A13, Purcell16, Caron18 and Peltier18, respectively. Similar to Fig. 7a, the gray arrow is the 1900–1978 average trend in PM observation, and the black arrow is the linear mean pole. All estimates are similar to one another and close to the linear mean pole (1.68 mas/year, − 3.46 mas/year). The linear mean pole is toward 64.10° W at a speed of 3.85 mas/year (11.88 cm/year), and our new estimate (mean of four estimates), (1.03 mas/year, − 1.92 mas/year), is in a direction 61.77° W at a speed of 2.18 mas/year (6.74 cm/year), smaller in magnitude but within the range of estimates based on rotational stability theory (Mitrovica and Wahr 2011). On the other hand, the average trend in PM observations for 1900–1978, (0.62 mas/year, − 3.48 mas/year), differs from our estimate, toward 79.90° W at a speed of 3.53 mas/year (10.91 cm/year).

## Discussion

Figure 7 shows that our new estimate of GIA PM trend is close to the linear mean pole (average PM trend for 1900–2017) but evidently different from model predictions which are closer to the observed trend for 1900–1978. Differences between our new estimate and GIA model predictions are possibly associated with other causes of a linear trend in PM such as mantle convection (Adhikari et al. 2018). If mantle convection is important, then our estimated GIA PM trend would include this effect. Thus the difference between GIA model predictions and PM observations could be taken as an estimate of the mantle convection contribution. Mantle convection effects on PM have been computed in a number of studies, but estimates have been quite variable (Adhikari et al. 2018).

Another plausible cause of the difference would be uncertainty in GIA models. The gray horizontal lines in Fig. 6b show PM trends for 1900–1978 which Wahr et al. (2015) assumed were due to GIA. In fact, these trends (gray lines) must be long-term means of contemporary surface mass load and GIA effects. Because GIA model predictions may be constrained by PM observation (Wahr et al. 2015), GIA predictions would be contaminated by contemporary surface mass load contributions. Blue horizontal lines in Fig. 6b show our new estimate of $\left( {\dot{m}_{1}^{{{\text{GIA}}}} ,\dot{m}_{2}^{{{\text{GIA}}}} } \right)$ from the mean of four different estimates in Fig. 7b. Figure 6b shows that our new estimate for the GIA PM trend is similar to the 40-year mean during the first half of the twentieth century. This is consistent with the hypothesis that GIA dominated PM trends during the early twentieth century and that later trends are more affected by contemporary surface mass redistribution, perhaps related to a warming climate. The agreement between our GIA PM trend and the current linear mean pole also supports this hypothesis. As shown in Fig. 6b, there are evident multi-decadal oscillations in the 40-year mean PM trends. Such oscillations are likely caused by surface mass load redistribution since 1900. An average PM trend over a fairly long period of time (over a century from 1900 to 2017) would likely suppress these multi-decadal oscillations. Because the contemporary sources (AIS, GrIS, MG and TWS) are geographically distinct, directions of associated PM trends are different, as clearly shown by the thin red arrows in Fig. 5b. Therefore, the different contributions may largely cancel one another over a sufficiently long period of time. However, in recent decades, GrIS and AIS have emerged as dominant contributors to contemporary surface mass load changes (Shepherd et al. 2018, 2020), so in the future, the linear mean pole trend would tend toward 0° E, deviating further from the GIA direction.

## Conclusion

Over the period 1900–1978 observed average PM drift is toward 79.90° W at a speed of 3.53 mas/year (10.91 cm/year). This has been assumed to be due to GIA, but it must also include effects from climate-driven contemporary surface mass loads and possibly mantle convection or other interior sources. GIA model predictions confirm its importance, but there are significant model-to-model differences, making it difficult to separate GIA from other causes. The linear mean pole, the average PM trend for 1900–2017 has been also assumed to be GIA PM and used in GRACE pole tide corrections. It shows an evidently different direction (64.10° W). We use GRACE gravity data to correct GIA models for contemporary surface load contributions by assuming that changes in SH coefficients above degree 2 are dominated by surface mass load effects, and that mass exchanges between land and oceans are associated with ocean mass distribution that conforms to SAL theory. Four different GIA models were corrected including A13, Purcell16, Caron18 and Peltier18. Estimated GIA PM based on the four corrected GIA models has a mean speed and direction of 2.18 mas/year (6.74 cm/year) and 61.77° W, respectively. The GIA PM direction estimated in this study is very close to the observed PM trend during the early twentieth century when surface mass load redistribution associated with climate warming was probably less important than GIA. Our new GIA PM also agrees with the linear mean pole during 1900–2017. Average PM trend over such a long period of time would suppress multi-decadal effects associated with climate changes and thus represent the GIA PM. However, in the future, the linear mean pole trend would differ from the GIA PM because the contributions from AIS and GrIS have emerged. A revised estimate of the GIA trend has implications for the processing of GRACE data; in particular, this would revise the correction made for the pole tide. GRACE data provide unique information in understanding causes of PM, and further contributions can be expected from GRACE-FO as the gravity field time series is extended.

## Data Availability

CSRRL06 GRACE data can be downloaded at ftp://podaac.jpl.nasa.gov/allData/grace/L2/. SLR ΔC20 and PGR model of A13 are available at GRACE Tellus Web site (https://grace.jpl.nasa.gov/data/get-data/). PGR model of Purcell17 can be downloaded from their Supporting Information (https://agupubs.onlinelibrary.wiley.com/action/downloadSupplement?doi=10.1002%2F2015JB012742&file=jgrb51576-sup-0003-supplementary.txt), Caron18 can be downloaded at JPL Web site (https://vesl.jpl.nasa.gov/solid-earth/gia/downloads/GIA_present_day_grid_file.txt), and Peltier18 can be accessed from Dr.Peltier’s Web site (http://www.atmosp.physics.utoronto.ca/~peltier/data.php). For construction of synthetic GRACE data, GLDAS data can be downloaded at Goddard Earth Sciences Data and Information Services Center (GES DISC; https://disc.gsfc.nasa.gov/datasets/GLDAS_NOAH10_M_001/summary?keywords=GLDAS), and glacier data are available from the Zenodo repository (https://doi.org/10.5281/zenodo.1492141). PM parameters are obtained from IERS (https://www.iers.org). The data that support the findings of this study will be available upon request.
